# Comparison of the therapeutic effects between stem cells and exosomes in primary ovarian insufficiency: as promising as cells but different persistency and dosage

**DOI:** 10.1186/s13287-023-03397-2

**Published:** 2023-06-20

**Authors:** Hang-soo Park, Rishi Man Chugh, Jin Seok, Esra Cetin, Hanaa Mohammed, Hiba Siblini, Farzana Liakath Ali, Mohammad Mousaei Ghasroldasht, Hiba Alkelani, Amro Elsharoud, Mara Ulin, Sahar Esfandyari, Ayman Al-Hendy

**Affiliations:** 1 Department of Obstetrics and Gynecology, University of Chicago, 5841 S. Maryland Ave., Chicago, IL 60637 USA; 2 Department of Surgery, University of Illinois at Chicago, Chicago, IL 60612 USA; 3 Department of Radiation Oncology, University of Kansas Medical Center, Kansas City, KS 66160 USA; 4 Human Anatomy and Embryology Department, Faculty of Medicine, Sohag University, Sohag, 82524 Egypt

**Keywords:** Mesenchymal stem cell, Exosome, Primary ovarian insufficiency, Infertility, Long-term effect

## Abstract

**Background:**

Primary ovarian insufficiency (POI) refers to the loss of ovarian function under the age of 40 and results in amenorrhea and infertility. Our previous studies have shown that transplantation of mesenchymal stem cells (MSCs) and MSC-derived exosomes in chemotherapy-induced POI mouse ovaries can reverse the POI and eventually achieve pregnancy. Based on our recent studies, MSC-derived exosomes have almost equal therapeutic potentials as transplanted MSCs. However, it is still unclear whether exosomes can completely replace MSCs in POI treatment. For the reliable application of cell-free treatment for POI patients using exosomes, there is a need to understand whether there is any outcome and effectiveness difference between MSC and MSC-derived exosome treatment.

**Methods:**

Comparing the therapeutic effect of intravenous injection using MSCs and equal amounts of exosomes in a POI mouse model will reveal the difference between the two therapeutic resources. In this study, we induced POI in C57/BL6 mice by chemotherapy (CXT) using a standard protocol. We then injected four different doses of MSCs or equal amounts of commercialized MSC-derived exosomes by retro-orbital injection post-CXT.

**Result:**

After MSC/exosome treatment, tissue and serum samples were harvested to analyze molecular changes after treatment, while other mice in parallel experiments underwent breeding experiments to compare the restoration of fertility. Both the MSC- and exosome-treated groups had a restored estrous cycle and serum hormone levels compared to untreated POI mice. The pregnancy rate in the MSC-treated group was 60–100% after treatment, while the pregnancy rate in the exosome-treated group was 30–50% after treatment. Interestingly, in terms of long-term effects, MSC-treated mice still showed a 60–80% pregnancy rate in the second round of breeding, while the exosome-treated group became infertile again in the second round of breeding.

**Conclusions:**

Although there were some differences in the efficacy between MSC treatment and exosome treatment, both treatments were able to achieve pregnancy in the POI mouse model. In conclusion, we report that MSC-derived exosomes are a promising therapeutic option to restore ovarian function in POI conditions similar to treatment with MSCs.

**Supplementary Information:**

The online version contains supplementary material available at 10.1186/s13287-023-03397-2.

## Background

Primary ovarian insufficiency (POI), also formerly known as premature ovarian failure (POF), refers to ovarian loss of function under the age of 40 and leads to menopausal symptoms and infertility [1–3]. There are various known risk factors for POI, such as genetic issues, autoimmune diseases, metabolic disorders, viral infections, chemotherapy, and radiotherapy exposure. Most of the POI cases are idiopathic, there is no identifiable risk factors in those cases [4], and the rest of cases are defined as iatrogenic POI. Chemotherapy exposure is the most common cause of iatrogenic POI, accounting for approximately half of the new cases of POI in women of reproductive age (20–80%). POI is frequently reported in cancer survivors after cure, and POI incidence has been increasing with the recent developments in chemotherapeutical agents [5, 6]. Most cancer patients receive chemotherapy to inhibit the growth of tumor cells. However, chemotherapy also causes apoptosis of ovarian cells, especially granulosa cells (GCs) which is essential cell for follicular development. Eventually, it leads to follicular atresia and decreased ovarian function, all of which lead to the clinical criteria of POI [7–9].

Because chemotherapy is a well-known risk factor for POI, the chemotherapy-induced POI model is well established, and many studies have used this model to develop POI treatment. Many published studies, including our previous papers, used a chemotherapy-induced POI mouse model to mimic human POI patients [10–12]. Healthy mice were used to establish a POI model by intraperitoneal injection of chemotherapies, such as cyclophosphamide and busulfan. Similar to human patients, the POI mouse model shows an arrested estrous cycle, increased serum FSH levels, decreased serum AMH levels, decreased ovary size (ovarian atrophy), and a low follicle numbers [10–12].

Recently, many studies have reported promising therapeutic effects of MSCs on POI treatment [13–17]. These studies have demonstrated that MSCs affect ovarian function in POI patients through different mechanisms, including homing, differentiation, and paracrine stimulation [18, 19]. The homing of transplanted MSCs to the ovary in POI animal models, which affects the successful transplantation of MSCs, has been mediated by different mechanisms and molecules [20–23]. Some studies have reported that human MSC-derived menstrual blood and skin were differentiated into GCs and ovarian stroma cells in an animal model of POI [8, 13, 24, 25]. However, most researchers believe the opposite opinion that MSCs do not differentiate into GCs because these cells do not express GC markers after transplantation [26–29].

In recent studies, it has been reported that the regenerative potential of MSCs is mainly attributed to paracrine effects and exosomes, which are small extracellular vesicles (EVs) secreted by cells [30]. MSCs can secrete multiple factors, including cytokines and exosomes, that result in POI recovery through several mechanisms, including reducing apoptosis and inflammation and inducing angiogenesis. Our previous study also showed that MSCs restore ovarian function by secreting factors such as cytokines, growth factors, and exosomes [10, 11]. In particular, exosomes have a great potential to deliver miRNAs or proteins to target cells without any concern about safety due to the fact that they are allogeneic cells. Exosomes are nanosized extracellular vesicles that work as messenger cargo for intercellular communication. Recent studies have indicated that MSC-derived exosomes can restore ovarian biological activity and show a similar therapeutic effect as the transplantation of MSCs [31–35]. It has been indicated that purified exosomes from different cell sources, including umbilical cord-derived MSCs, menstrual blood-derived stromal cells, and adipose-derived MSCs, rejuvenate chemotherapy-induced damage by reducing GC apoptosis, inducing GC proliferation, inhibiting follicle apoptosis, promoting follicle development, and regulating various pathways. Moreover, exosomes restore estrous cyclicity and hormone levels and improve reproductive outcomes in POI animal models [31–35].

MSC-derived exosomes offer many useful properties compared to those of whole live cell transplantation. Cell therapy manufacturing is an expensive and complex process, and multiple parameters, such as the identity, purity, potency, stability, and potency of the products, must be closely monitored throughout the manufacturing process. The challenges of the storage, delivery, and shipping of cell products make the process harder, even though it is still considered an effective and promising treatment approach for a variety of diseases [36]. Therefore, exosomes can be an excellent alternative treatment modality since they offer less expensive, more accessible, yet effective, ready-to-use treatment options for regenerative medicine [37, 38]. In addition, exosomes do not cause genomic integration due to the lack of a nucleus and show regulatory ability with targeting cells through the high loading capacity of proteins and RNAs [39]. Considering these findings, exosome therapy is currently becoming the most superior and cutting-edge treatment option in regenerative disorders. Exosomes derived from MSCs are expected to replace stem cells as a new therapeutic option for regeneration as a stem cell-based cell-free approach [40]. However, there is a need for further studies, such as comparing with live MSCs and understanding the mechanisms that contribute to tissue repair and regenerative properties, before using exosome therapy in the clinic. In particular, it is still unclear whether exosomes can completely replace MSCs in future POI treatment. For example, exosomes may degrade much earlier than MSCs in in vivo conditions and may affect therapeutic outcomes.

The field of regenerative medicine has been extensively exploring and utilizing stem cells in recent years. Nevertheless, the risk of tumorigenicity, genomic insertion of transgenic sequences, and low efficiency have raised concerns, especially for clinical applications of embryonic stem cells (ESCs) and pluripotent stem cells (iPSCs) [41]. Mesenchymal stem cells (MSCs), on the other hand, have shown immune-responsive modulation with immunosuppressive properties, yet the risk of tumorigenesis associated with MSC therapy is under debate [42]. Recently, exosomes derived from MSCs have attracted immediate attention due to their wide availability and accessibility, as they are secreted by various cell types [43–45]. Moreover, exosomes offer an excellent feasible alternative, as they are more accessible and less expensive to obtain than the tedious process of preparing and storing stem cells [37, 38]. Considering this, exosome therapy is being widely applied and studied in regenerative medicine clinical applications as a more suitable, safer, stem cell-based cell-free approach [46, 47].

Due to many benefits, we mentioned earlier that using exosomes instead of MSCs to restore ovarian function is a very promising option in the future in the clinic. For the reliable application of cell-free treatment for POI patients using exosomes, there is a need to understand whether there is any outcome difference between MSC and MSC-derived exosome treatment. In this study, we compared the therapeutic effect of intravenous injection using MSCs and equal amounts of exosomes in a POI mouse model and revealed the difference between the two therapeutic resources.

## Methods

- hBM-MSC cell culture and exosome preparation

Human bone marrow mesenchymal stem cells (hBM-MSCs) were purchased from Roosterbio (Frederick, MD); these cells were isolated from the bone marrow of a 29-year-old female and a 26-year-old female. hBM-MSCs were cultured in the recommended cell culture media, RoosterNourish™-MSC-XF (Roosterbio). At approximately 80% confluence, the cells were trypsinized using CTSTM TrypLE select enzyme (Gibco, MA) and were serially expanded for two additional passages. At the end of the culture expansion, hBM-MSCs were collected and centrifuged at 300 × *g* for 5 min. For intravenous injection, 1 × 10^4^, 1 × 10^5^, and 1 × 10^6^ hBM-MSCs were resuspended in 100 µl of phosphate-buffered saline (PBS).

Human MSC-derived exosomes were a kind gift from the Vitti laboratory (Liberty, MO) and Direct Biologics (Austin, TX). One product (Ev-pure, Vitti lab) was isolated from umbilical cord MSCs, and the other product (ExoFlo, Direct Biologics) was derived from bone marrow MSCs. The manufacturer (Vitti Lab) indicated that approximately 1500 exosomal particles were produced from one MSC over 24 h. Based on this manufacturer’s information, 1.5 × 10^7^, 1.5 × 10^8^, and 1.5 × 10^9^ exosomal particles were resuspended in 100 µl of PBS for animal study.

- POI mouse model

The experimental animal protocol in this study was approved by the University of Illinois at Chicago Animal Care Committee (UIC ACC) and the University of Chicago Institutional Animal Care and Use Committee (UC IACUC). All animal experiments were performed in accordance with the ethical policy and guidelines for the use of laboratory animals as set by the University of Illinois at Chicago and the University of Chicago. Author Checklist for preclinical study was assessed based on ARRIVE guidelines 2.0 (Additional file 1). We used a chemotherapy-induced POI animal model and an hBM-MSC intravenous injection protocol through retro-orbital injection. Briefly, female C57BL/6 mice received an intraperitoneal injection of busulfan (30 mg/kg) and cyclophosphamide (120 mg/kg). The control group was treated with PBS. After 7 days of chemotherapy, hBM-MSCs were transplanted into the ovary via intravenous injection. All mice were anesthetized using 1–4% isoflurane inhalation. A total of 1 × 10^4^ to 1 × 10^6^ hBM-MSCs were resuspended in 100 µl of PBS and injected through the retro-orbital sinus. For exosome injection, 1.5 × 10^7^ to 1.5 × 10^9^ exosomal particles were resuspended in 100 µl of PBS and injected through the same route. For the control and untreated POI groups, each mouse was injected with 100 µl of PBS through intravenous injection. Seven days after hBM-MSC or exosome treatment, two female mice were housed with one C57BL/6 male mouse for breeding. The pregnancy rate per group was calculated as the number of pregnant mice/total number of mice in the group. After delivery, postnatal pup body weight was measured at Days 0, 5, and 10. For blood collection, animals were anesthetized with isoflurane, and collect whole blood through cardiac puncture. After blood collection animals were euthanized for further tissue collection. All animals were euthanized by CO2 for 5 min before other tissue collection. Only specimens were released out of the animal facility.

- Estrous cycle monitoring

Estrous cycles were monitored daily for 14 days after the initiation of chemotherapy to verify chemotherapy-induced POI. Daily vaginal swab samples were collected using clean and sterile cotton swabs and smeared onto clean glass slides for staining, which were then evaluated to determine the estrous cycle stage. Each slide was stained with 0.1% crystal violet solution for 1 min following a published protocol [48]. The estrous cycle stage was evaluated using bright-field microscopy based on the presence or absence of nucleated epithelial cells and leukocytes [48, 49]. Arrested cycles were analyzed based on 95% confidence interval (95% CI) of control group based on length of entire cycle. Animals show longer cycle than 95% CI of matched control group considered significant extended cycle which indicates cycle arrest.

- Histology

Ovarian tissue was collected 2 weeks after hBM-MSC or exosome treatment for histological analysis. Ovaries were fixed immediately with 10% neutral buffered formalin (NBF) and then processed for paraffin embedding, sectioning, and staining for hematoxylin and eosin staining (H&E) and the tunnel assay in the UIC Research Histology and Tissue Imaging Core and University of Chicago HTRC. For quantification of stained slides, whole-stained slides were scanned using a Leica Aperio AT2 camera (Leica, Wetzlar, Germany) and analyzed using Aperio ImageScope software (v12.4.0.5043). In each image, the ovarian tissue area was selected manually, and the positivity rate (positive area/total area) was analyzed in the selected area by an internal algorithm (Positive Pixel Count V9). To analyze the collagenic structure in the ovary, we performed picrosirius red staining (in-house protocol). Paraffin-embedded tissue sections were deparaffinized and incubated in picrosirius red solution (Abcam) at RT for 1 h. Then, the slides were quickly washed with 1% acetic acid and 100% absolute alcohol and mounted in mounting solution. Images were captured using a CRi Pannoramic SCAN 40× whole slide scanner (3D HISTECH), and the medulla part in the ovarian tissue was selected. The total collagen content was determined for the medulla images using ImageJ software. For safety tests in other major organs, we collected the liver, lung, and spleen. The organs were processed and stained with H&E as described above and examined for any pathological damage (inflammation and abnormal structure).

- RNA sequencing

RNA isolation was performed using an RNeaxy Micro-kit (Qiagen, Hilden, Germany) according to the manufacturer’s instructions. The RNA concentration was quantified by spectrophotometry at 260 nm using a Nanodrop 2000 (Thermo Fisher Scientific). Approximately 1–1.5 µg of RNA sample was sent to the core facility (University of Chicago Genomics Facility) for further processing. RNA-seq was performed by the Genomic facility with NovaSEQ-SP-100-FC (Illumina, San Diego, CA) and analyzed by bioinformation in the CRI Bioinformatics core facility at the University of Chicago. The entire data set is available in the NCBI gene expression omnibus (GEO) database (GSE233743, https://www.ncbi.nlm.nih.gov/geo/query/acc.cgi?acc=GSE233743).

- Human cell detection by genomic DNA PCR

For genomic DNA isolation, 25–50 mg of mouse tissue was mechanically homogenized with 1 ml of DNAzol reagent (Thermo Fisher Scientific). The homogenate was centrifuged at 10,000× *g* for 10 min at room temperature, and the supernatant was transferred into a fresh tube. Then, 0.5 ml of 100% ethanol was added to the supernatant for DNA precipitation. Genomic DNA was isolated following the manufacturer’s protocol. The concentration of DNA was quantified using a Nanodrop 2000 (Thermo Fisher Scientific). For human cell detection by PCR, a human-specific ALU primer sequence was used as described in the literature [50]. Genomic DNA PCR was performed with ALU primers under the following conditions: one cycle of 95 °C for 10 min, followed by 50 cycles of 95 °C for 15 s, 56 °C for 30 s, and 72 °C for 30 s. The number of human cells in each sample was calculated using the correlation between the CT value and cell number in the positive control sample.

- Serum hormone measurements

Blood was collected from all the groups by cardiac puncture under isoflurane anesthesia; serum was separated by centrifugation at 2000× *g* for 15 min at room temperature and stored at − 80 °C. Serum hormone levels were measured at the University of Virginia Ligand Core Facility. Serum estradiol (E2) levels were measured using ELISA. Serum luteinizing hormone (LH) and follicle-stimulating hormone (FSH) levels were measured by radioimmunoassay (RIA). The sensitivities of each assay were 3 pg/ml (E2), 3 ng/ml (FSH), and 0.04 ng/ml (LH).

- Statistical analysis

The mRNA and protein levels of the examined markers were treated as continuous variables and expressed as the mean ± SD. ANOVA and Bonferroni’s multiple comparisons post hoc testing were used to compare the groups. Comparisons between groups were made by two-way ANOVA using GraphPad Prism 9 (GraphPad Software, San Diego, CA, USA). A difference between groups of *p* < 0.05 was considered significant. For the comparison of estrus cycle, extended cycle was analyzed based on 95% confidence interval (95% CI) of control group. Animals show longer cycle than 95% CI upper limit of matched control group considered significant extended cycle. The upper and lower limit of 95% CI was calculated using GraphPad Prism 9 (GraphPad Software, San Diego, CA, USA).

## Results

- Intravenous injection of 10,000 MSCs can restore the estrous cycle

To generate a POI mouse model, female C57BL6 mice were treated with busulfan (30 mg/kg) and cyclophosphamide (120 mg/kg) by intraperitoneal injection.

**Fig. 1:**
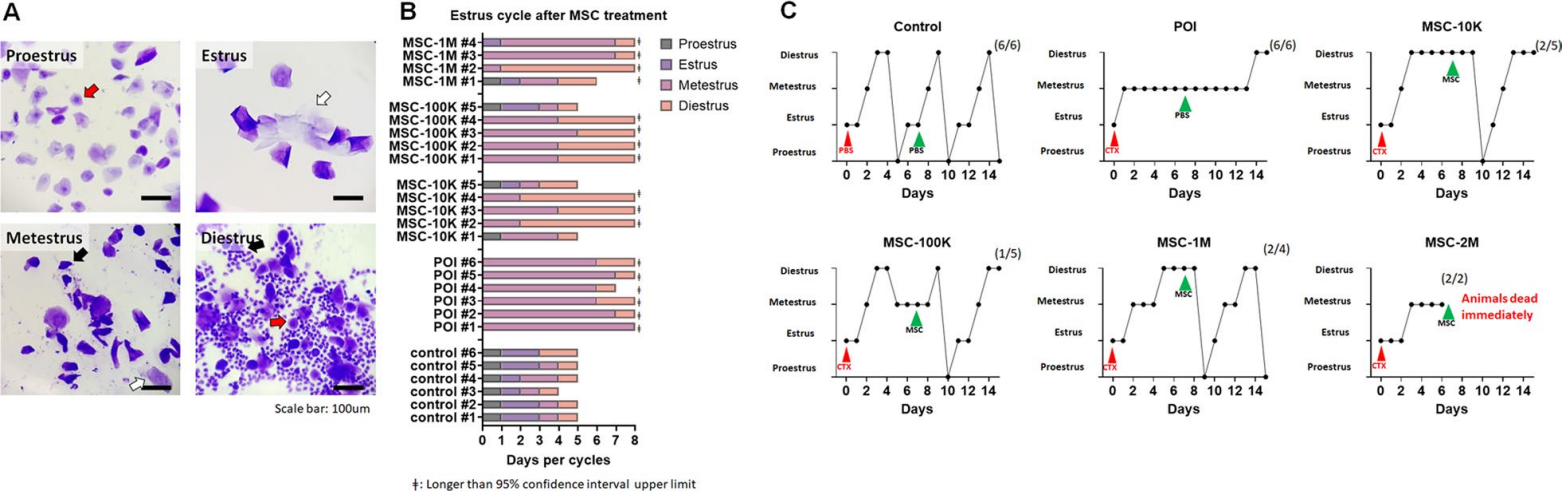
Restored estrous cycle in the MSC-injected POI mouse model. Three different numbers of MSCs (MSC-10K: 10,000 cells/100 µL, MSC-100K: 100,000 cells/100 µL, and MSC-1M: 1,000,000 cells/100 µL) were injected intravenously. **A** Representative image of the proestrus, estrus, metestrus, and diestrus phases in mouse vaginal smear samples. Animal shows significantly delayed cycle (> 95% CI) is highlighted with ǂ symbol. **B** The estrous cycle length in each mouse after MSC treatment. **C** Representation of the estrous cycle change in mice by daily vaginal smear analysis

We injected different numbers of MSCs to optimize the best treatment conditions. A minimum of 10,000 MSCs (MSC-10 K) and a maximum of 2,000,000 MSCs (MSC-2M) were injected through retro-orbital intravenous (IV) injection. Unfortunately, mice in the MSC-2M group died immediately due to excess condensed solution. The daily identification of estrous cycle stages through bright-field microscopy of vaginal smear samples was analyzed together to confirm POI induction and restoration of ovarian function. Based on the protocol of published papers [12, 48], the presence of nucleated epithelial cells (red arrow) without leukocytes was identified as proestrus (Fig. 1a upper-left), the presence of cornified epithelial cells (white arrow) without leukocytes as estrus (Fig. 1a upper-right), the presence of both cornified epithelial cells (white arrow) and leukocytes (black arrow) as metestrus (lower-left), and the presence of nucleated epithelial cells (red arrow) with leukocytes (block arrow) as the diestrus stage (lower-right). Our results showed an arrested estrous cycle in the POI group, while untreated control mice showed a regular 4- to 5-day estrous cycle (Fig. 1b, c, Additional file 3). All MSC-treated groups showed a restored estrous cycle compared to the untreated POI group. Among different numbers of treatment conditions, the MSC-10K group showed the same restoration effect even when the lowest number of MSCs was used for injection. As a result, we used 10,000 MSCs to qualify the therapeutic effect of intravenous MSC injection and performed a subsequent comparison study.

- Intravenous injection of MSCs restores ovarian function

**Fig. 2:**
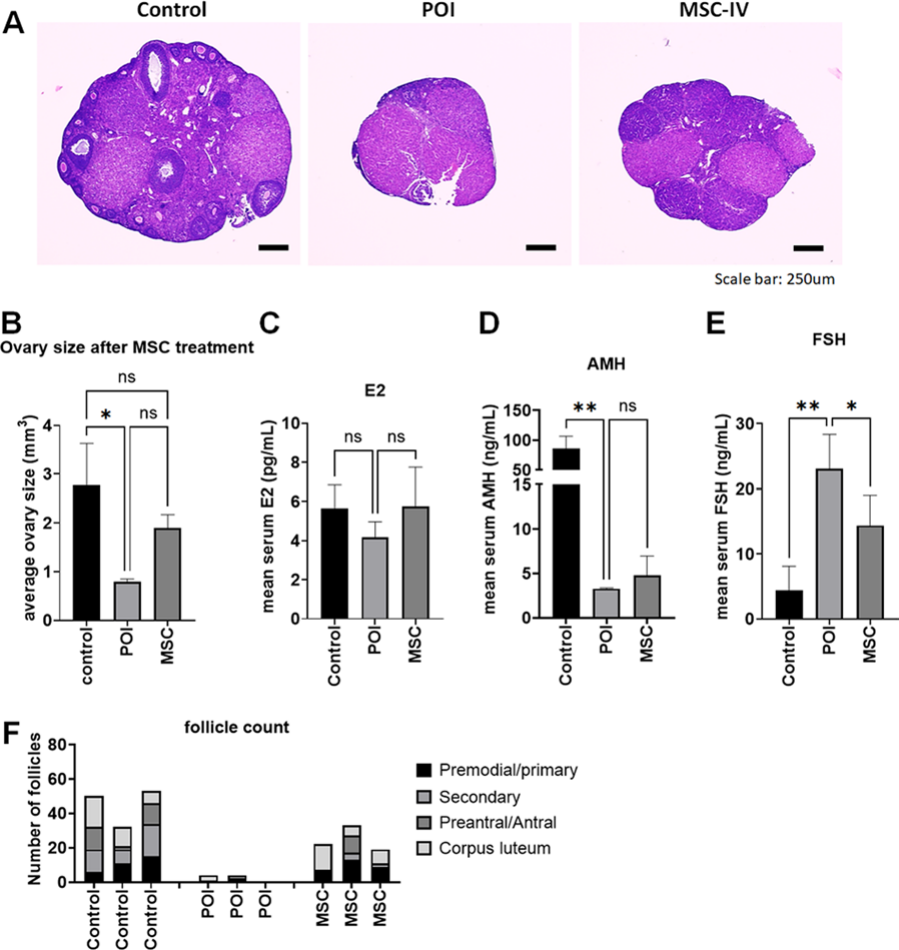
Restored ovarian function by MSC treatment. **A** Representative mouse ovarian tissue image stained with H&E. **B** Average size of the mouse ovaries in healthy mice (control), untreated POI mice (POI), and MSC-treated POI mice (MSC) (n = 3 per group). **C**–**E** Average serum E2, AMH, and FSH levels in the control, POI, and MSC groups (n = 3 per group). **F** Number of ovarian follicles in the control, POI, and MSC groups. Primordial/primary follicles, secondary follicles, preantral/antral follicles, and corpus lutea were differentially counted and merged into one stacked bar (n = 3 per group)

As we observed that IV injection of MSCs can restore the estrous cycle, we analyzed more detailed outcomes with respect to ovarian histology and serum hormone levels as a next step. For ovarian histology, ovarian tissue section slides were processed for H&E staining. We measured the size of the ovary and the number of follicles to qualify the therapeutic effect and further compare the results. We observed that the size of the ovary was significantly (*p* = 0.013) decreased in the POI group (0.79 ± 0.06 mm^3^) compared to the healthy control group (2.77 ± 0.86 mm^3^), while there was no significant change (*p* = 0.134) between the healthy control and MSC-treated groups (MSC-IV, 1.90 ± 0.27 mm^3^) (Fig. 2a, b). At the serum hormone level, we analyzed estradiol (E2), anti-Müllerian hormone (AMH), and follicle stimulating hormone (FSH) to assess ovarian function (Fig. 2c–e). The serum E2 level showed a decreasing trend in POI mice (4.18 ± 0.79 pg/ml) that was reversed after MSC treatment (5.75 ± 2.00 pg/ml) but was not significant (Fig. 2c). The serum AMH level was significantly decreased in POI mice (3.29 ± 0.08 ng/ml) and showed an increasing trend in MSC-treated mice (4.78 ± 2.19 ng/ml) but was not significant (Fig. 2d). The FHS level was significantly increased in POI mice (23.08 ± 5.26 ng/ml) compared to control mice (4.38 ± 3.75 ng/ml). Interestingly, altered FSH levels were significantly restored in MSC-treated mice (14.39 ± 4.88 ng/ml) (Fig. 2e). Taken together, we found that intravenous injection of MSCs successfully restored ovarian function in a POI mouse model.

We also found that IV injection of MSCs can regulate RNA expression levels in ovarian tissue.

- Intravenous injection of MSCs restores fertility

**Fig. 3:**
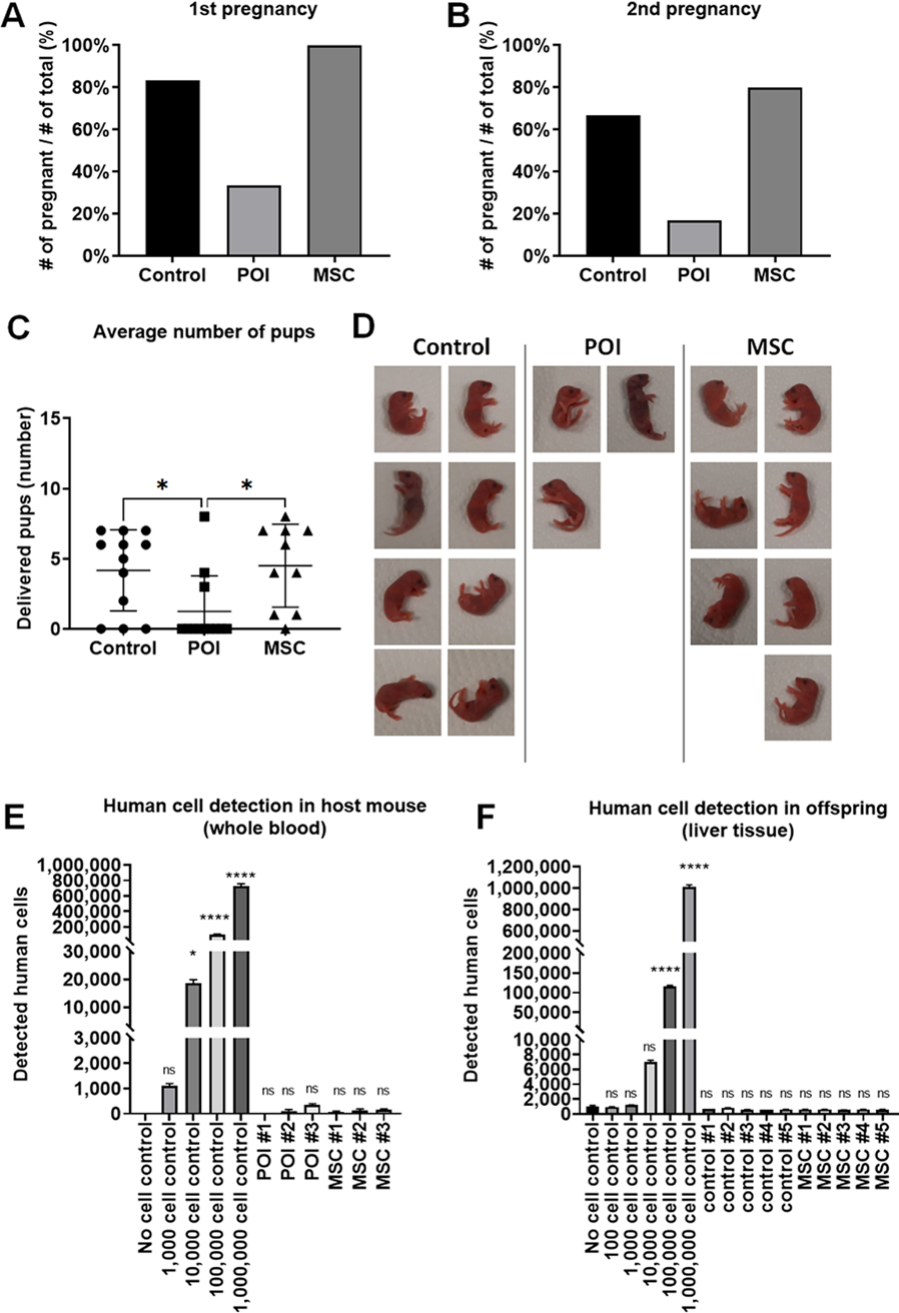
Fertility was restored by MSC treatment in the POI mouse model (n = 6 per group). **A** Pregnancy rate comparison between healthy mice (control), untreated POI mice (POI), and MSC-treated POI mice (MSC) in the first breeding experiment. **B** Pregnancy rate comparison between the control group, POI group, and MSC group in the second breeding experiment. **C** Average number of delivered pups per litter. **D** Representative image of delivered pups at Day 0. **E** Genomic DNA PCR analysis for detecting injected cells (human MSCs) in mouse whole blood using human-specific ALU primers (n = 3 per group). **F** Genomic DNA PCR analysis for detecting injected cells in neonatal offspring liver tissue using human-specific ALU primers (n = 5 per group). Data are presented as the mean ± SD. (Significance level, *p < 0.05, **p < 0.005, ***p < 0.0005, ****p < 0.0001; NS: Not significant.)

To confirm the effect on fertility, we performed a breeding experiment. Two female mice were housed with one male mouse for mating. In the first breeding test, the POI mouse group showed a decreased pregnancy rate (33.3%), while healthy control mice showed an 83.3% pregnancy rate. Interestingly, every female mouse in the MSC-treated group was pregnant and delivered healthy offspring (Fig. 3a). After the first delivery, we repeated the breeding experiment and found similar results (Fig. 3b). Most MSC-treated mice were able to become pregnant (80%), although it was approximately 30 days after MSC treatment. On the other hand, only a few mice in the POI group were pregnant (16.67%). The number of pups also significantly decreased in POI mice and was significantly restored in MSC-treated mice (Fig. 3c). The average number of pups per mouse from the first and second breeding was 4.17 ± 2.89 in the healthy control group, 1.25 ± 2.53 in the POI group, and 4.50 ± 2.95 in the MSC-treated group. After the second delivery, we collected whole blood from the mice to examine safety concerns, such as the remaining human cells in the blood. We isolated genomic DNA from mouse blood and analyzed the existence of a human-specific ALU gene by PCR. We found that no human cells were detected in MSC-treated mouse blood (Fig. 3e). We also analyzed pup tissue to examine the possibility of the transfer of human cells to the mouse offspring. As we expected, human DNA was not detected in mouse offspring tissue. Taken together, we demonstrate that intravenous injection of MSCs successfully restored fertility without safety issues.

- Therapeutic effect of exosomes in human granulosa cells

**Fig. 4:**
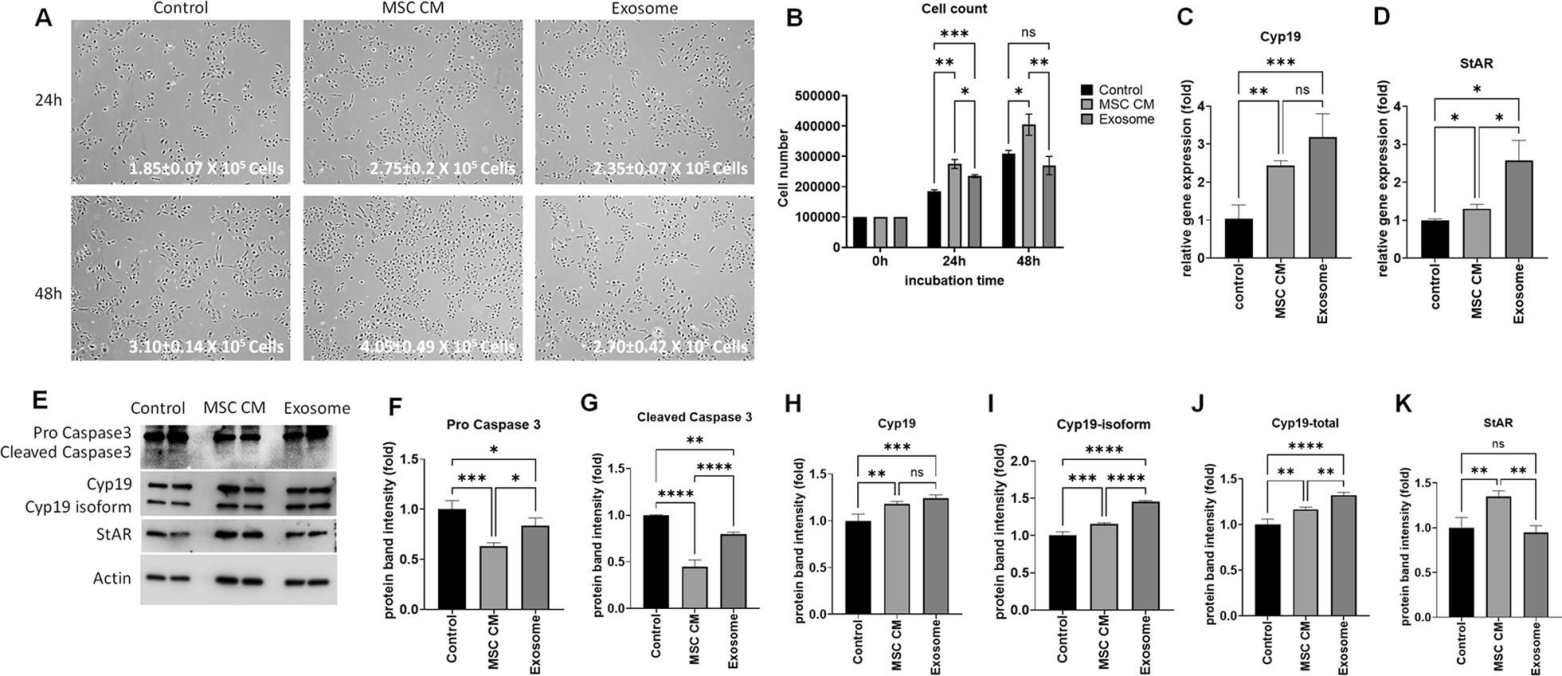
Effect of MSC-derived exosomes in an in vitro POI model on damaged human granulosa cells (HGrC1). The therapeutic effect in HGrC1 cells was compared between untreated control (control), MSC conditioned media treatment (MSC CM), and MSC-derived exosome treatment (exosomes). **A** Morphology of HGrC1 cells in the control, MSC CM, and exosome groups after 24 h and 48 h of treatment. **B** Average number of HGrC1 cells after MSC CM and exosome treatment. **C**, **D** Steroid gene expression levels (C: Cyp19, D: StAR) in HGrC1 cells after 24 h of treatment with MSC CM and exosomes. **E**–**K** Protein expression levels of the apoptosis marker caspase-3 (**E**–**G**) and steroidogenesis markers (**I**–**K**) in western blot analysis. Data are presented as the mean ± SD. (n = 3, significance level, *p < 0.05, **p < 0.005, ***p < 0.0005, ****p < 0.0001; NS: Not significant.)

Before we tested the therapeutic effect of MSC-derived exosomes in the POI mouse model, we analyzed exosomes in our in vitro POI model. We used cyclophosphamide-treated human granulosa cells (hGCs) to mimic chemotherapy-induced POI in patients. In this study, we used commercially available exosomes derived from umbilical tissue MSCs. Based on the manufacturer’s QC datasheet, 1500 exosomal particles were secreted from one MSC. We used 1.5 × 10^9^ exosomal particles (produced from 1 × 10^6^ MSCs) and 5 ml of MSC conditioned media (MSC CM) produced from 1 × 10^6^ MSCs. To compare the effect of MSC conditioned media (MSC CM) and exosomes in hGCs, we cultured hGCs with MSC CM and exosomes for 24–48 h and then collected hGCs to analyze proliferation and steroidogenesis gene expression. We found that the number of hGCs after 24 h was significantly higher in the MSC CM-treated group (2.75 ± 0.2 × 10^5^) and exosome-treated group (2.35 ± 0.07 × 10^5^) compared to the control group (1.85 ± 0.07 × 10^5^). Interestingly, after 48 h of treatment, the number of hGCs in the exosome-treated group (2.70 ± 0.42 × 10^5^) was not higher than that of the control group (3.10 ± 0.14 × 10^5^), while the MSC CM-treated group (4.05 ± 0.49 × 10^5^) still showed increased hGC proliferation (Fig. 4a, b). This result suggested that the therapeutic effect of exosomes was similar to that of total MSC conditioned media within 24 h, but the effect was nullified at approximately 48 h. Because we found that exosomes showed a similar effect as total MSC CM at 24 h, we collected hCG RNA and proteins after 24 h of incubation for further analysis. In real-time RT‒PCR analysis, we analyzed the steroidogenesis expression of marker genes such as Cyp19a1 and STAR. We found that Cyp19a1 gene expression was significantly increased in MSC CM-treated hGCs (2.43 ± 0.13-fold) and exosome-treated hGCs (3.18 ± 0.61-fold). STAR gene expression also increased in MSC CM-treated hGCs (1.30 ± 0.12-fold) and exosome-treated hGCs (2.58 ± 0.53-fold). As a next step, we analyzed apoptosis markers and steroidogenesis markers at the protein level by Western blotting (Fig. 4e–k). Our Western blot results showed that the apoptosis marker cleaved caspase-3 was significantly decreased in MSC CM-treated hGCs (0.45 ± 0.07-fold) compared to untreated control hGCs (1.00 ± 0.01-fold). Exosome-treated hCG also showed decreased cleaved caspase-3 levels (0.79 ± 0.02-fold) compared to the control level but higher levels than MSC CM-treated hGCs (Fig. 4e, f). The levels of the steroidogenesis marker Cyp19a1 were also significantly increased in both MSC CM-treated hGCs (1.17 ± 0.03-fold) and exosome-treated hGCs (1.32 ± 0.03-fold) compared to control hGCs (Fig. 4h–j), as we observed at the RNA expression level. On the other hand, another steroidogenesis marker, STAR, was significantly increased in MSC CM-treated hGCs (1.35 ± 0.07-fold) but was not changed in exosome-treated hGCs (0.95 ± 0.08-fold) compared to untreated hGCs (Fig. 4k). Taken together, our results suggest that MSC-derived exosomes have therapeutic effects similar to those of total MSC conditioned media on damaged human granulosa cells, including the stimulation of proliferation and steroidogenesis marker gene expression and inhibition of apoptosis.

- Therapeutic effect of exosomes in the POI mouse model

**Fig. 5:**
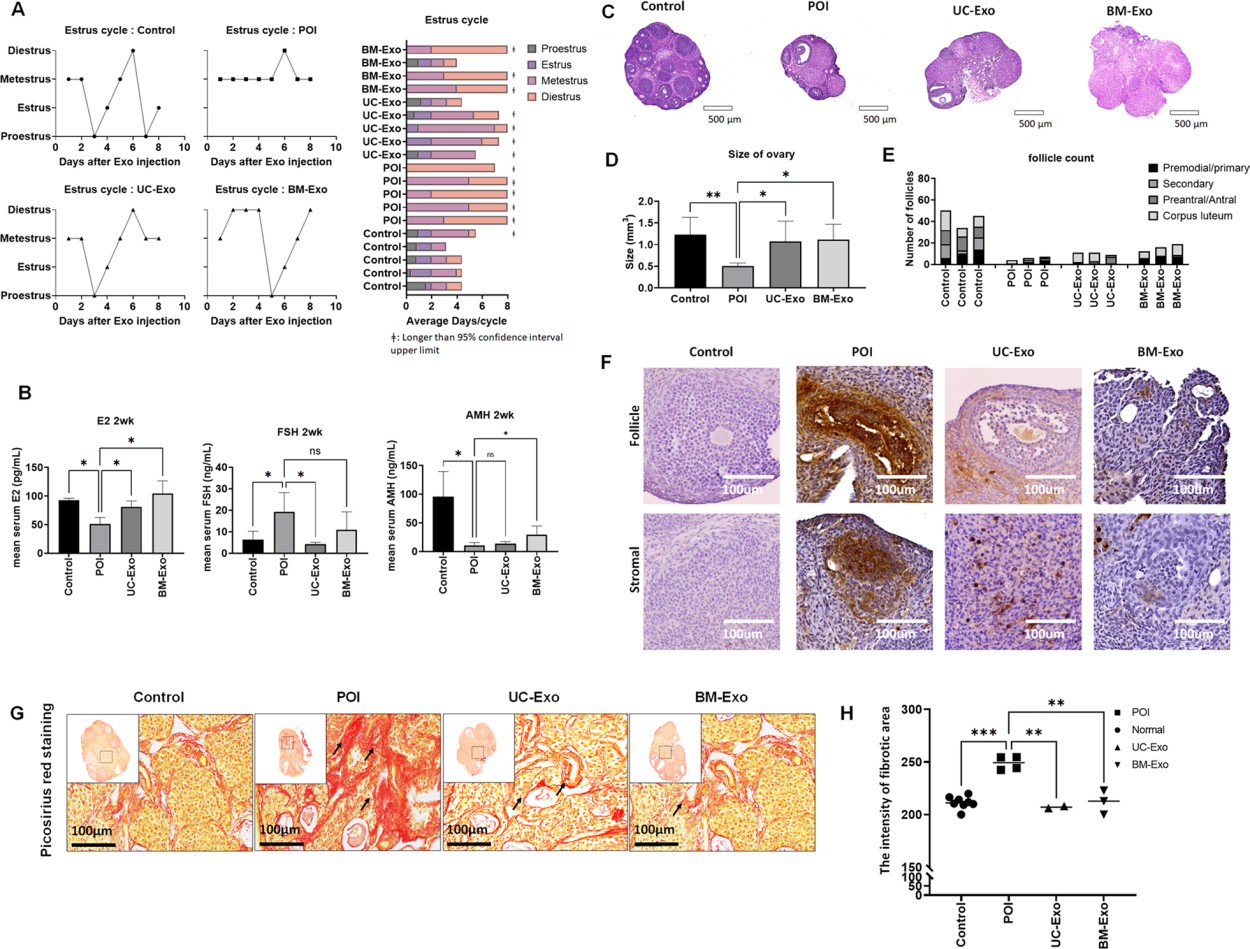
Ovarian function restoration in the POI mouse model after exosome treatment. The therapeutic effect in the POI mouse model was compared between the healthy control (control), untreated POI (POI), umbilical-derived MSC exosome treatment (UC-Exo), and bone marrow-derived MSC-derived exosome treatment (BM-Exo) groups. **A** Estrous cycle after exosome treatment by daily vaginal smear analysis. Animal shows significantly delayed cycle (> 95% CI) is highlighted with ǂ symbol. **B** Average serum E2, AMH, and FSH levels in the control, POI, UC-Exo, and BM-Exo groups at 2 weeks after treatment. **C** Representative image of ovarian tissue with H&E staining. **D** Average size of the ovaries in the control, POI, UC-Exo, and BM-Exo groups at 2 weeks after treatment. **E** Number of ovarian follicles in the control, POI, UC-Exo, and BM-Exo groups at 2 weeks after treatment. **F** TUNEL assay in ovarian tissue among the control, POI, UC-Exo, and BM-Exo groups at 2 weeks after treatment. **G**, **H** Picrosirius Red staining assay in ovarian tissue among the control, POI, UC-Exo, and BM-Exo groups at 2 weeks after treatment (small image magnification: 50×; large image magnification: 400×). Data are presented as the mean ± SD. (n = 3, significance level, *p < 0.05, **p < 0.005, ***p < 0.0005, ****p < 0.0001; NS: Not significant.)

After we confirmed the therapeutic effect of exosomes in an in vitro model, we tested the effect of exosomes in a POI mouse model. Similar to the MSC experiment, we used a chemotherapy-induced POI mouse model and retro-orbital IV injection. To inject equal amounts of exosomes compared to our previous experiment using MSCs, we used 1.5 × 10^7^ exosomal particles (produced from 1 × 10^4^ MSCs). To confirm any difference due to the origin of exosomes, we tested both exosomes derived from umbilical tissue MSCs (UC-Exos) and exosomes derived from bone marrow MSCs (BM-Exos). As a first step in analyzing the therapeutic effect of exosomes in the POI mouse model, we compared the estrous cycle between the healthy mouse group (control), POI mouse group (POI), UC-Exo-treated POI mouse group (UC-Exo), and BM-Exo-treated POI mouse group (BM-Exo). We found that the estrous cycle was restored in several mice after exosome treatment (Fig. 5A, Additional file 3). Next, we analyzed the serum levels of hormones, such as E2, AMH, and FSH, at 2 weeks after exosome treatment (Fig. 5B). We found that serum E2 levels were significantly restored in both UC-Exo-treated mice (80.95 ± 10.45 pg/ml) and BM-Exo-treated mice (104.7 ± 21.75 pg/ml), while untreated POI mice showed decreased serum E2 levels (51.00 ± 11.50 pg/ml) compared to those of healthy control mice (92.65 ± 3.75 pg/ml). FSH levels, which are typically increased in POI mouse serum (19.27 ± 8.90 ng/ml), were also significantly restored in UC-Exo-treated mice (4.18 ± 0.91 ng/ml). The BM-Exo-treated mice also showed a decreasing trend in FSH levels (10.86 ± 8.39 ng/ml), but this trend was not significant (*p* = 0.163). In contrast, the serum AMH level was significantly restored in BM-Exo-treated mice (29.30 ng/ml), while UC-Exo-treated mice (13.41 ± 4.80 ng/ml) did not show a significant change compared to the levels of untreated POI mice (10.53 ± 4.88 ng/ml). We also collected mouse ovarian tissue to compare the histological differences between POI mice and exosome-treated mice. In H&E staining of mouse ovaries, we found that the size of the ovary was significantly restored in both UC-Exo-treated mice (1.11 ± 0.36 mm^3^) and BM-Exo-treated mice (1.07 ± 0.47 mm^3^), while POI mice showed reduced ovarian size (0.50 ± 0.07 mm^3^) compared to that of healthy mouse ovaries (1.22 ± 0.40 mm^3^). The number of follicles was also restored in both UC-Exo-treated mice and BM-Exo-treated mice compared to untreated POI mice. In the TUNEL assay, we found a higher TUNEL-positive population in POI mouse ovaries, which indicates apoptosis in ovarian tissue. In contrast, there was a smaller TUNEL-positive population in both UC-Exo- and BM-Exo-treated ovaries, which indicates reduced apoptosis in ovarian tissue. The characteristic of collagen accumulation in ovarian tissue is ovarian fibrosis, which is one of the main causes of ovarian dysfunction and indicates excessive proliferation of ovarian fibroblasts and deposition of extracellular matrix (ECM) [51]. Hence, to analyze ovarian fibrosis, we performed picrosirius red staining in ovarian tissues. As shown in Fig. 5E, we confirmed that the red color representing accumulated collagen significantly increased in untreated POI mice. In contrast, in the ovarian tissue injected with UC-Exos and BM-Exos, collagen accumulation was significantly reduced, indicating a value similar to that of healthy control mice (Fig. 5G, H). Taken together, our results demonstrated that exosome treatment can restore ovarian function in a POI mouse model.

- Fertility restoration by exosomes in the POI mouse model

**Fig. 6:**
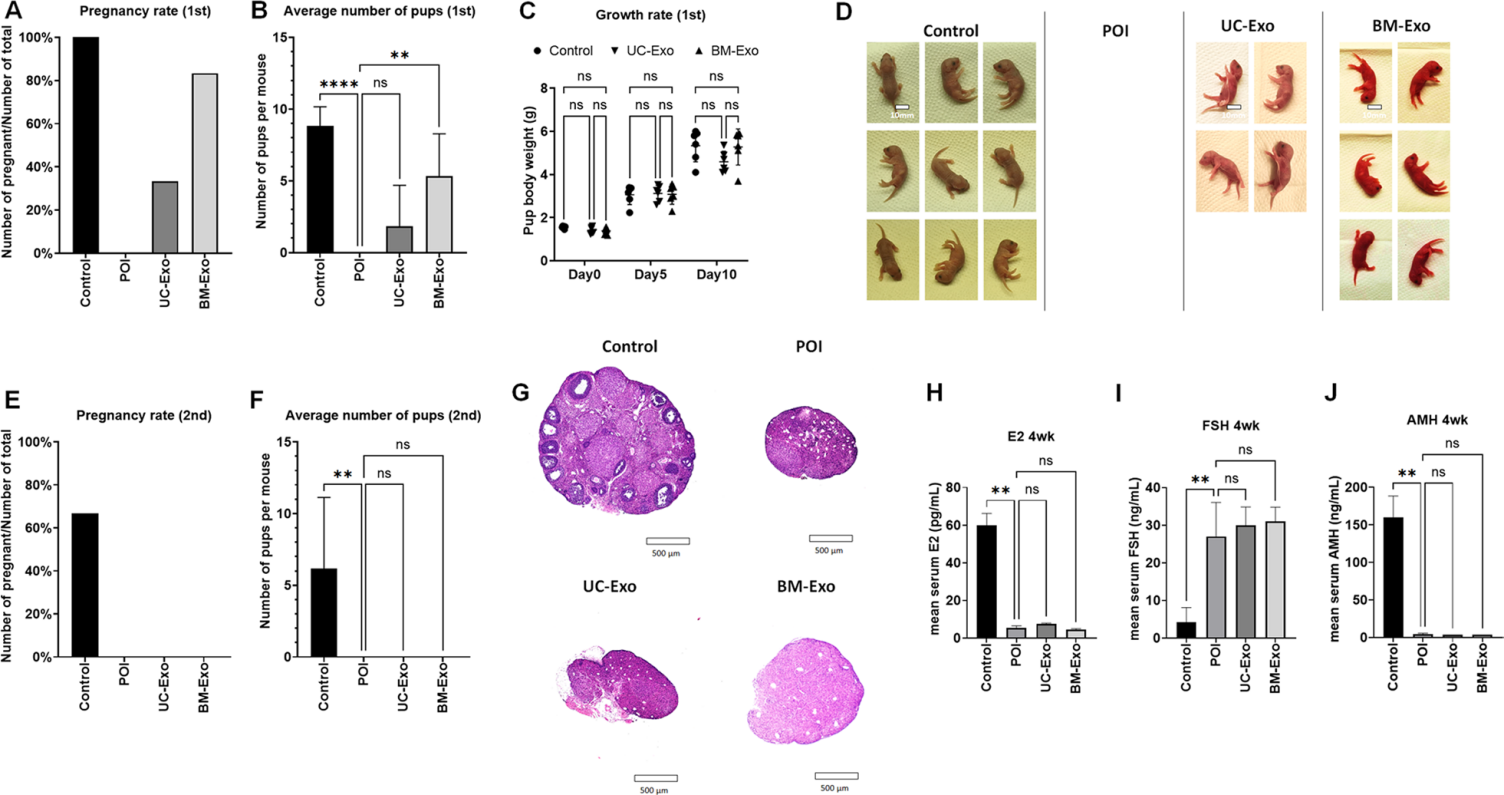
Fertility restoration in the POI mouse model after exosome treatment. **A** Pregnancy rate in the healthy control (control), untreated POI (POI), umbilical-derived MSC exosome treatment (UC-Exo), and bone marrow-derived MSC-derived exosome treatment (BM-Exo) groups in the first breeding. **B** Average number of pups per litter between the control, POI, UC-Exo, and BM-Exo groups in the first breeding. **C** Postnatal growth rate comparison between the control, UC-Exo, and BM-Exo groups. **D** Representative image of delivered pups on Day 0. **E** Pregnancy rate of the control, POI, UC-Exo, and BM-Exo groups in the second breeding. **F** Average number of pups per litter between the control, POI, UC-Exo, and BM-Exo groups in the second breeding. **G** Ovarian tissue morphology after the second breeding. **H**–**J** Serum hormone levels after the second breeding. Data are presented as the mean ± SD. (n = 3, significance level, *p < 0.05, **p < 0.005, ***p < 0.0005, ****p < 0.0001; NS: not significant.)

To confirm the therapeutic effect of exosomes in the POI mouse model, we compared the fertility and delivery outcomes in untreated POI mice and exosome-treated mice. In the breeding experiment, we analyzed the pregnancy rate and number of delivered pups to assess fertility and compared the average body weight until postnatal Day 10 to evaluate the safety. In our first breeding experiment, we found that both exosome-treated groups showed a restored pregnancy rate (2/6 in UC-Exos, 4/6 in BM-Exos), while untreated mice were not pregnant (0/6) (Fig. 6A, B). The exosome-treated groups delivered a minimum of 4 to a maximum of 8 pups per litter, while healthy control mice delivered approximately 7–11 pups per litter. We also confirmed that the delivered pups were healthy and active with no morphological deficiency (Fig. 6C, D). All delivered pups grew well until postnatal Day 10, and there was no significant difference in pup body weight between the healthy control group (5.33 ± 0.75 g), UC-Exo group (4.59 ± 0.49 g), and BM-Exo group (5.27 ± 0.84 g). After we confirmed the healthy delivery in exosome-treated POI mice, we repeated the breeding to confirm the persistence of exosome treatment. The second breeding was performed approximately one month after exosome treatment. Interestingly, we found that the treatment effect of exosomes was nullified in the second breeding. Both exosome-treated groups (UC-Exos and BM-Exos) were infertile, similar to untreated POI mice (Fig. 6E, F). Because there were no pregnant mice in either treated mouse group, we killed those mice for further molecular-level analysis. To analyze mice under the same conditions, we allowed 2 weeks of recovery time after the last delivery to healthy control group mice before collecting tissue and serum samples. We analyzed the morphology of ovarian tissue in those mice by H&E staining. We found that the exosome-treated mouse ovaries, which showed a restoration of their size and follicle number, exhibited POI characteristics again, including a small ovarian size and no follicles (Fig. 6G). We also analyzed serum hormone levels, such as E2, FSH, and AMH, which were once restored at 2 weeks after treatment. Our results showed that all of these serum hormones were reversed to the POI condition at this point. There was no significant difference in the E2 levels of the POI group (5.43 ± 1.08 pg/ml), UC-Exo group (7.53 ± 0.38 pg/ml) and BM-Exo group (4.57 ± 0.40 pg/ml). The FSH level also did not show any difference between the POI group (26.98 ± 9.04 ng/ml), UC-Exo group (29.88 ± 4.96 ng/ml), and BM-Exo group (31.05 ± 3.74 ng/ml). The serum AMH level also indicated that there was no difference between the POI group (4.01 ± 1.53 ng/ml), UC-Exo group (3.25 ± 0.01 ng/ml), and BM-Exo group (3.24 ± 0.01 ng/ml). Taken together, our data suggest that exosome treatment can restore fertility in a POI mouse model in the short term, but the therapeutic effect disappeared approximately one month later.

- Comparison of MSC and exosome treatment for POI

**Fig. 7:**
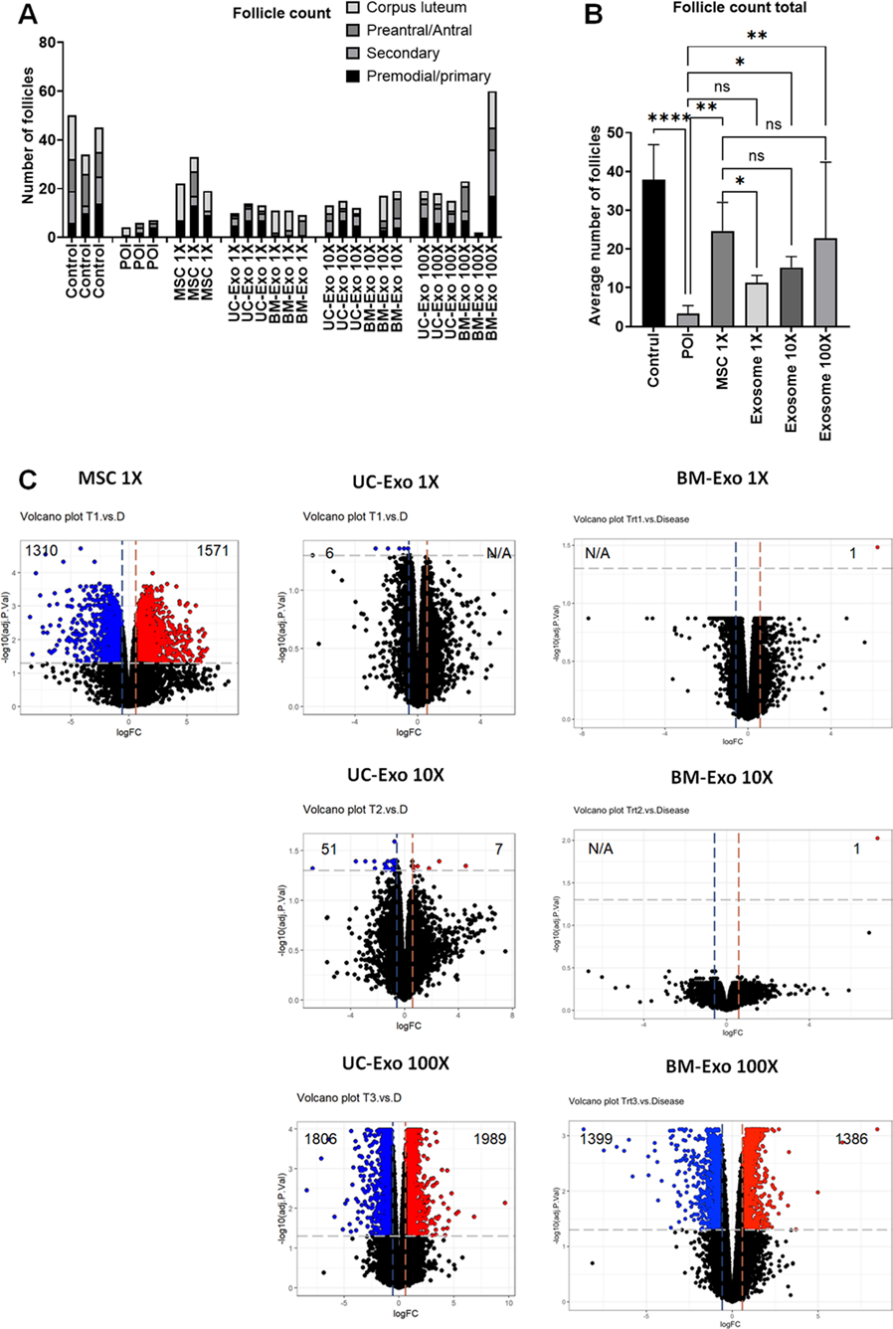
Differences between MSC treatment and exosome treatment in the POI mouse model. **A** Number of ovarian follicles in the healthy control (control), untreated POI group (POI), MSC treatment group (MSC 1×), equal amount of exosomes (UC-Exo 1×, BM-Exo 1×), tenfold higher amount of exosomes treatment (UC-Exo 10×, BM-Exo 10×), and 100-fold higher amount of exosomes treatment (UC-Exo 100×, BM-Exo 100X). **B** Comparison of the average number of total follicles among the control, POI, MSC 1×, average UC-Exo and BM-Exo (exosomes 1×, 10×, and 100×) groups. **C** Altered gene expression by RNA-seq analysis in each treatment compared with untreated POI mouse ovaries. A volcano plot shows significantly increased genes (blue) and significantly decreased genes (red) after MSC and exosome treatment. Data are presented as the mean ± SD. (n = 3, significance level, *p < 0.05, **p < 0.005, ***p < 0.0005, ****p < 0.0001; NS: Not significant.)

As we observed through our data, MSC and MSC-derived exosomes were able to restore fertility in the POI mouse model. However, exosome treatment was effective for only a limited period, while MSC treatment showed a long-term therapeutic effect. In addition to the persistency of treatment, we also found that there was another difference in efficacy between MSC treatment and exosome treatment. Comparing the number of follicles in the ovary, we found that the efficacy of an equal amount of exosomes was slightly lower than that of MSCs (Fig. 7A, B). To normalize estrous cycle-dependent variation, we compared the total number of follicles, including primordial, primary, secondary, preantral/antral follicles, and corpus lutea. In our data, we found that an equal amount of exosomes (exosome 1×) showed significantly fewer follicles (11.33 ± 1.86) than MSC treatment (MSC 1×, 24.67 ± 7.37), although both were able to achieve pregnancy in the mouse model, while untreated POI mice were infertile.

To find a better dosage for exosome treatment that was comparable to our optimal MSC treatment dosage (10,000 cells), we injected higher amounts of exosome particles (tenfold and 100-fold) into the POI model and analyzed ovarian follicles. Interestingly, we found that tenfold higher exosomes (exosome 10×) show higher follicle numbers (15.20 ± 2.86), and there was no significant difference with the MSC 1× group. When we injected 100-fold more exosome particles (exosome 100×), the number of follicles showed an increasing trend (22.83 ± 19.57) compared to the exosome 10× group, but there was no significant difference (*p* = 0.204) (Fig. 7B). Next, we compared the RNA expression pattern in ovarian tissue for further analysis (Fig. 7C, Additional file 4). In our RNA-seq results, we found that MSC injection (MSC 1×) in the POI mouse model induced massive gene expression changes in ovarian tissue. In the MSC 1× group, the ovaries showed 1310 significantly upregulated genes (blue) and 1571 significantly downregulated genes (red) compared to POI ovarian RNA. In contrast, equal amounts of UC exosomes (UC-Exo 1×) and BM exosomes (BM-Exo 1×) showed only less than 10 significantly altered genes compared to untreated POI. Ten-fold higher exosome treatment showed slightly higher altered gene expression in UC exosomes (UC-Exo 10×) but not in BM exosomes (BM-Exo 10×). Interestingly, when we injected 100-fold higher exosomes (UC-Exo 100× and BM-Exo 100×), we found almost equal altered gene expression levels compared to that of MSCs (MSC 1×). Taken together, our data suggest that a minimal ten-fold increase, and ideally 100-fold increase, in the exosome concentration is required to generate more analogous results to MSC treatment.

## Discussion

In this study, we reported that MSC-derived exosomes have promising therapeutic potential compared to that of MSCs. Despite some concern for the low activity of cell-free treatment using exosomes, our data show that intravenous injection of exosomes can restore ovarian function and eventually achieve pregnancy in a POI mouse model. However, there are some different outcomes between live MSC and exosome treatments. The efficacy is slightly higher with MSCs, and the effect is sustained longer than that with exosomes. However, exosomes were also able to achieve pregnancy and were still superior with respect to safety. Therefore, we suggest that POI treatment using exosomes is safe and promising but effective only in a limited period due to the low stability of exosomes after injection. Our study revealed that exosome treatment is still promising, but the sustainability of treatment is shorter than that of MSCs. Therefore, we suggest that the multidose regimen in exosome treatment is essential for maintaining therapeutic effects in the long term.

After MSC/exosome treatment, tissue and serum samples were harvested to analyze molecular changes after treatment without pregnancy. Mice in separate parallel experiments were subjected to breeding experiments to compare the effect of restoring fertility in this preclinical POI model (6 mice/group). For the MSC-treated groups, mice received a low dose (1.0 × 10^4^ MSCs), mid dose (1.0 × 10^5^ MSCs), and high dose (1.0 × 10^6^ MSCs) through intravenous injection. For the exosome-treated groups, mice received a low dose (1.5 × 10^7^ particles [equal to 1.0 × 10^4^ MSCs]), mid-dose (1.5 × 10^8^ particles [equal to 1.0 × 10^5^ MSCs]), and high dose (1.5 × 10^9^ particles [equal to 1.0 × 10^6^ MSCs]). Because we made separate experimental groups and breeding groups using the same procedure, we analyzed biological marker changes (experimental group) and fertility (breeding group) without any interference due to sample collection and pregnancy.

In this study, we used a chemotherapy-induced POI mouse model with cyclophosphamide and busulfan [11, 12, 52]. Unlike human patients who receive chemotherapy, a few mice can still become pregnant even after chemotherapy, while most mice are infertile after chemotherapy. However, although our POI mouse model was not 100% infertile, we observed significant fertility restoration after MSC and exosome treatment compared to untreated POI mice.

We also have a limitation in the animal model analysis due to the small sample size in the serum hormone assay. We compared hormone levels using three independent mouse serum samples per each group, but this result might be limited to generalize it due to the small sample size. Unfortunately, we could not analyze more animal samples due to limited resources and technical difficulties in sample collection. However, although our serum analysis has limitations, we showed another parameter to quantify restored fertility, such as ovary morphology, estrus cycle, and eventually the pregnancy in the mouse model. Therefore, taking them altogether, our data still demonstrate that MSCs and exosome treatment in the POI model can restore fertility.

Chemotherapy is not the only reason for POI in human patients. A recent study of the etiological factors in 827 POI patients reported that most POI patients have idiopathic diseases, such as autoimmune diseases, for which the cause is unknown. However, in other cases with iatrogenic factors, chemotherapy and radiotherapy were some of the most frequent risk factors in iatrogenic POI patients (35.9%), except for direct damage to the ovary due to surgery [53]. Other studies also reported that chemotherapy is one of the well-verified causes of POI, while other risk factors are very rare or are unknown [54, 55]. Therefore, we used a chemotherapy-induced POI mouse model in our study. Similar to chemotherapy, radiotherapy can be an interesting model to develop potential treatment options for POI. It would be an interesting topic for our future studies to confirm and verify the therapeutic mechanism of MSCs and exosomes in damaged ovaries using several similar models.

In our study, we found that the effect of exosome treatment was shorter than that of MSC treatment. Although MSC injection was effective for a longer period of time, we previously reported that the effect of MSCs also decreased approximately 70 days after treatment [12]. On the other hand, the effect of exosomes disappeared much earlier, approximately 30 days after treatment, even though they maintained restored ovarian function 14 days after treatment. This result can be explained by the short half-life of exosomes in vivo. In a published paper, it was reported that more than half of injected exosomes were removed from the blood circulation within 60 min after intravenous injection. They also reported that the exosome detection level in mouse urine was maximized between 60 and 90 min after intravenous injection [56]. Another published paper also reported that all exosomes from different sources quickly disappeared from the blood circulation, i.e., there was less than half within approximately 2–4 min. The authors reported that injected exosomes were mainly distributed to the liver after intravenous injection into mice [57]. Another recent study reported that the mean residence time of injected exosomes in various mouse models was less than 4 h [58]. These published studies indicate that injected exosomes disappear very rapidly in vivo. In addition, due to the nature of exosomes, injected exosomes are not reproducible once they are taken up by the target cell. On the other hand, injected cells can keep secreting exosomes while the cells survive. These characteristics of exosomes may explain different outcomes regarding the long-term effects between MSC and exosome treatment.

In this study, we showed that RNA expression profile changes in POI ovary after MSC/exosome treatment. Gene expression changes were presented as volcano plots with log fold change (LogFC) and P-value. By comparing untreated disease samples with treated samples (T vs. D), negative LogFC means lower gene expression in disease samples, which indicates stimulated gene expression after treatment. The list of significantly changed genes is attached in Additional file 4. In our previous study, we reported that MSC can stimulate steroidogenic genes such as Cyp19a1 and StAR gene in ovarian granulosa cell [11]. We also found that StAR gene was significantly stimulated in MSC treated ovary tissue. However, StAR gene was not significantly stimulated in exosome treated ovary tissue. Unlike our previous study using granulosa cell, the whole ovary consisted with multiple cell population. Due to heterogenic population, it is not easy to analyze the regulation pathway for specific type of cells in whole tissue sample. Confirming the main regulation pathway can be analyzed with more advanced techniques such as single cell sequencing might be considered in future study to reveal the detailed mechanism.

The low efficacy of exosome treatment compared to matched-dose MSC treatment could be considered a limitation of exosome treatment. However, our data show that a higher concentration of exosomes can enhance the therapeutic effect to a level similar to that of MSC treatment. In addition, cell-free therapy using secreting factors, including exosomes, has various benefits compared to live cell transplantation. In published papers, it has been reported that cell-free treatment provides several advantages over live-cell-based applications, including low immune response, lack of tumorigenicity, and no embolus formation, and can be evaluated for safety, dosage, and potency in a manner similar to conventional pharmaceutical agents [59]. In addition, this type of treatment can be stored without cryopreservation agents and is easy to apply for mass production [59]. Therefore, exosome treatment still has a safety benefit as a cell-free treatment, and the low efficacy can be overcome by high-dose treatment.

Many current and completed clinical trials have already reported the therapeutic benefits of exosomes in various conditions, which have been registered in the www.ClinicalTrials.gov database [47, 60]. Kordelas et al. demonstrated the efficacy and safety of allogeneic bone marrow mesenchymal stem cell-derived exosomes in therapy-refractory graft-versus-host disease. Their findings were extremely encouraging in terms of exosome treatment’s anti-inflammatory and proinflammatory cytokine capabilities. After repeated injections of MSC-EVs, patients' cutaneous and mucosal graft-versus-host disease symptoms improved significantly, and the patients remained stable for several months after the injection [61]. Nassar et al. used umbilical cord-derived exosomes for end-stage renal disease (grade III-IV CKD); they administered cell-free cord-blood mesenchymal stem cell extracellular vesicles through intravenous injection and renal artery injection one week apart. Their study did not show any adverse events after exosome injection. Patients showed a significant improvement in kidney function, and this clinical finding was consistent with immune modulation; there was a significant decrease in TNF-α levels and a significant increase in TGF-β1 and IL-10 levels after treatment. According to their findings, exosome treatment has favorable effects similar to those of stem cell therapy [62]. Kwon et al. completed a double-blind, randomized, first-in-human clinical trial to treat acne scars and assess the potential benefits of purified adipose tissue stem cell-derived exosomes following fractional CO2 laser irradiation in 25 patients. They demonstrated the favorable effects of adjuvant therapy of adipose tissue stem cell-derived exosomes with resurfacing devices on atrophic acne scars [63]. Most recently, during the COVID-19 pandemic, multiple trials were conducted with the aim to take advantage of the therapeutic and anti-inflammatory effects of exosome treatment in severe COVID-19 cases since there is no proven treatment modality yet [64–66]. Vik et al. showed the safety and efficacy of intravenous bone marrow mesenchymal stem cell-derived exosome treatment in severe COVID-19 patients. Patients received a single 15-mL intravenous dose of exosome treatment, and they evaluated both safety and efficacy from Days 1–14 posttreatment. There were no adverse events within 72 h of exosome administration. Exosomes derived from bone marrow mesenchymal stem cells were administered intravenously at a dose of 1 mL diluted in 100 mL of normal saline. Primary outcomes demonstrate that exosome therapy is safe, accessible, effective, and feasible [66]. Mitrani et al. [67] administered human amniotic fluid-derived nanoparticles intravenously in hospitalized severe COVID-19 patients, and they confirmed the safety and therapeutic effects of exosomes for respiratory complications in COVID-19 cases. Our study provides preliminary baseline data comparing MSC treatment and exosome treatment in a POI mouse model. Similar to other conditions, our next step will be a clinical trial to verify the therapeutic effect of MSC-derived exosomes in human POI patients.

## Conclusion

In conclusion, we reported that MSC-derived exosomes are promising therapeutic options to restore ovarian function under POI conditions. Exosome injection can restore ovarian function and fertility in a mouse model without any significant side effects in host mice and delivered offspring. Despite the short shelf life of exosomes, our data suggest that exosome treatment is not inferior compared to whole MSC treatment. In addition, our study indicates that the short shelf life after injection can be a benefit for safety purposes without affecting function.

Nevertheless, there are some different outcomes between MSC and exosome treatment. The efficacy is slightly higher with MSCs, and the effect is sustained longer than that with exosomes. This low efficiency of exosomes can be overcome by increasing the dosage or using repeated injections in future studies. Taken together, our data suggest that POI treatment using MSC-derived exosomes is safe and promising but effective only in a limited period due to the low stability of exosomes after injection.

## Supplementary Information

**Additional file 1.** Supplementary data1_Estrus cycle analysis for 8 days after MSC treatment.

**Additional file 2.** Supplementary data2_List of differentially expressed genes.

**Additional file 3.** Original blot images.

**Additional file 4.** ARRIVE author check list.

## Data Availability

The entire data set is available in the NCBI gene expression omnibus (GEO) database (GSE233743, https://www.ncbi.nlm.nih.gov/geo/query/acc.cgi?acc=GSE233743).

## Abbreviations

hBM-MSC: Human bone marrow-derived mesenchymal stem cell
POI: Primary ovarian insufficiency
CTX: Chemotherapy
IHC: Immunohistochemistry
PCR: Polymerase chain reaction
FBS: Fetal bovine serum
PBS: Phosphate-buffered saline
UIC ACC: University of Illinois at Chicago Animal Care Committee
UC IACUC: University of Chicago Institutional Animal Care and Use Committee
E2: Estradiol
LH: Luteinizing hormone
FSH: Follicle-stimulating hormone
AMH: Anti-Müllerian hormone
H&E: Hematoxylin–eosin
CI: Confidence interval

## References

[1] Beck-Peccoz P, Persani L (2006). Premature ovarian failure. Orphanet J Rare Dis.

[2] Christin-Maitre S, Braham R (2008). General mechanisms of premature ovarian failure and clinical check-up. Gynecol Obstet Fertil.

[3] Panay N, Fenton A (2008). Premature ovarian failure: a growing concern. Climacteric.

[4] Elfayomy AK, Almasry SM, El-Tarhouny SA, Eldomiaty MA (2016). Human umbilical cord blood-mesenchymal stem cells transplantation renovates the ovarian surface epithelium in a rat model of premature ovarian failure: possible direct and indirect effects. Tissue Cell.

[5] Blumenfeld Z, Evron A (2016). Endocrine prevention of chemotherapy-induced ovarian failure. Curr Opin Obstet Gynecol.

[6] Molina JR, Barton DL, Loprinzi CL (2005). Chemotherapy-induced ovarian failure: manifestations and management. Drug Saf.

[7] Blumenfeld Z (2012). Chemotherapy and fertility. Best Pract Res Clin Obstet Gynaecol.

[8] Lai D, Wang F, Dong Z, Zhang Q (2014). Skin-derived mesenchymal stem cells help restore function to ovaries in a premature ovarian failure mouse model. PLoS ONE.

[9] Morgan S, Anderson RA, Gourley C, Wallace WH, Spears N (2012). How do chemotherapeutic agents damage the ovary?. Hum Reprod Update.

[10] Park HS, Ashour D, Elsharoud A, Chugh RM, Ismail N, El Andaloussi A, Al-Hendy A (2019). Towards cell free therapy of premature ovarian insufficiency: human bone marrow mesenchymal stem cells secretome enhances angiogenesis in human ovarian microvascular endothelial cells. HSOA J Stem Cells Res Dev Ther.

[11] Park HS, Chugh RM, El Andaloussi A, Hobeika E, Esfandyari S, Elsharoud A, Ulin M, Garcia N, Bilal M, Al-Hendy A (2021). Human BM-MSC secretome enhances human granulosa cell proliferation and steroidogenesis and restores ovarian function in primary ovarian insufficiency mouse model. Sci Rep.

[12] Park HS, Chugh RM, Elsharoud A, Ulin M, Esfandyari S, Aboalsoud A, Bakir L, Al-Hendy A (2021). Safety of intraovarian injection of human mesenchymal stem cells in a premature ovarian insufficiency mouse model. Cell Transplant.

[13] Abd-Allah SH, Shalaby SM, Pasha HF, El-Shal AS, Raafat N, Shabrawy SM, Awad HA, Amer MG, Gharib MA, El Gendy EA (2013). Mechanistic action of mesenchymal stem cell injection in the treatment of chemically induced ovarian failure in rabbits. Cytotherapy.

[14] Guo JQ, Gao X, Lin ZJ, Wu WZ, Huang LH, Dong HY, Chen J, Lu J, Fu YF, Wang J (2013). BMSCs reduce rat granulosa cell apoptosis induced by cisplatin and perimenopause. BMC Cell Biol.

[15] Mohamed SA, Shalaby SM, Abdelaziz M, Brakta S, Hill WD, Ismail N, Al-Hendy A (2018). Human mesenchymal stem cells partially reverse infertility in chemotherapy-induced ovarian failure. Reprod Sci.

[16] Seok J, Park H, Choi JH, Lim JY, Kim KG, Kim GJ (2020). Placenta-derived mesenchymal stem cells restore the ovary function in an ovariectomized rat model via an antioxidant effect. Antioxidants (Basel).

[17] Terraciano P, Garcez T, Ayres L, Durli I, Baggio M, Kuhl CP, Laurino C, Passos E, Paz AH, Cirne-Lima E (2014). Cell therapy for chemically induced ovarian failure in mice. Stem Cells Int.

[18] Huang QY, Chen SR, Chen JM, Shi QY, Lin S (2022). Therapeutic options for premature ovarian insufficiency: an updated review. Reprod Biol Endocrinol.

[19] Zhang S, Zhu D, Mei X, Li Z, Li J, Xie M, Xie HJW, Wang S, Cheng K (2021). Advances in biomaterials and regenerative medicine for primary ovarian insufficiency therapy. Bioact Mater.

[20] Jalalie L, Rezaie MJ, Jalili A, Rezaee MA, Vahabzadeh Z, Rahmani MR, Karimipoor M, Hakhamaneshi MS (2019). Distribution of the CM-Dil-labeled human umbilical cord vein mesenchymal stem cells migrated to the cyclophosphamide-injured ovaries in C57BL/6 Mice. Iran Biomed J.

[21] Ling L, Hou J, Liu D, Tang D, Zhang Y, Zeng Q, Pan H, Fan L (2022). Important role of the SDF-1/CXCR4 axis in the homing of systemically transplanted human amnion-derived mesenchymal stem cells (hAD-MSCs) to ovaries in rats with chemotherapy-induced premature ovarian insufficiency (POI). Stem Cell Res Ther.

[22] Liu J, Zhang H, Zhang Y, Li N, Wen Y, Cao F, Ai H, Xue X (2014). Homing and restorative effects of bone marrow-derived mesenchymal stem cells on cisplatin injured ovaries in rats. Mol Cells.

[23] Song D, Zhong Y, Qian C, Zou Q, Ou J, Shi Y, Gao L, Wang G, Liu Z, Li H (2016). Human umbilical cord mesenchymal stem cells therapy in cyclophosphamide-induced premature ovarian failure rat model. Biomed Res Int.

[24] Manshadi MD, Navid S, Hoshino Y, Daneshi E, Noory P, Abbasi M (2019). The effects of human menstrual blood stem cells-derived granulosa cells on ovarian follicle formation in a rat model of premature ovarian failure. Microsc Res Tech.

[25] Noory P, Navid S, Zanganeh BM, Talebi A, Borhani-Haghighi M, Gholami K, Manshadi MD, Abbasi M (2019). Human menstrual blood stem cell-derived granulosa cells participate in ovarian follicle formation in a rat model of premature ovarian failure in vivo. Cell Reprogram.

[26] Ling L, Feng X, Wei T, Wang Y, Wang Y, Wang Z, Tang D, Luo Y, Xiong Z (2019). Human amnion-derived mesenchymal stem cell (hAD-MSC) transplantation improves ovarian function in rats with premature ovarian insufficiency (POI) at least partly through a paracrine mechanism. Stem Cell Res Ther.

[27] Ling L, Feng X, Wei T, Wang Y, Wang Y, Zhang W, He L, Wang Z, Zeng Q, Xiong Z (2017). Effects of low-intensity pulsed ultrasound (LIPUS)-pretreated human amnion-derived mesenchymal stem cell (hAD-MSC) transplantation on primary ovarian insufficiency in rats. Stem Cell Res Ther.

[28] Wang Z, Wang Y, Yang T, Li J, Yang X (2017). Study of the reparative effects of menstrual-derived stem cells on premature ovarian failure in mice. Stem Cell Res Ther.

[29] Zheng Q, Fu X, Jiang J, Zhang N, Zou L, Wang W, Ding M, Chen H (2019). Umbilical cord mesenchymal stem cell transplantation prevents chemotherapy-induced ovarian failure via the NGF/TrkA pathway in rats. Biomed Res Int.

[30] Keshtkar S, Azarpira N, Ghahremani MH (2018). Mesenchymal stem cell-derived extracellular vesicles: novel frontiers in regenerative medicine. Stem Cell Res Ther.

[31] Huang B, Lu J, Ding C, Zou Q, Wang W, Li H (2018). Exosomes derived from human adipose mesenchymal stem cells improve ovary function of premature ovarian insufficiency by targeting SMAD. Stem Cell Res Ther.

[32] Li Z, Zhang M, Zheng J, Tian Y, Zhang H, Tan Y, Li Q, Zhang J, Huang X (2021). Human umbilical cord mesenchymal stem cell-derived exosomes improve ovarian function and proliferation of premature ovarian insufficiency by regulating the hippo signaling pathway. Front Endocrinol (Lausanne).

[33] Sun L, Li D, Song K, Wei J, Yao S, Li Z, Su X, Ju X, Chao L, Deng X (2017). Exosomes derived from human umbilical cord mesenchymal stem cells protect against cisplatin-induced ovarian granulosa cell stress and apoptosis in vitro. Sci Rep.

[34] Zhang S, Huang B, Su P, Chang Q, Li P, Song A, Zhao X, Yuan Z, Tan J (2021). Concentrated exosomes from menstrual blood-derived stromal cells improves ovarian activity in a rat model of premature ovarian insufficiency. Stem Cell Res Ther.

[35] Zhang X, Zhang R, Hao J, Huang X, Liu M, Lv M, Su C, Mu YL (2022). miRNA-122-5p in POI ovarian-derived exosomes promotes granulosa cell apoptosis by regulating BCL9. Cancer Med.

[36] de Almeida Fuzeta M, de Matos Branco AD, Fernandes-Platzgummer A, da Silva CL, Cabral JMS, Silva AC, Moreira JN, Lobo JMS, Almeida H (2020). Addressing the manufacturing challenges of cell-based therapies. Current applications of pharmaceutical biotechnology.

[37] Toh WS, Lai RC, Hui JH, Lim SK (2017). MSC exosome as a cell-free MSC therapy for cartilage regeneration: implications for osteoarthritis treatment. Semin Cell Dev Biol.

[38] Adamiak M, Sahoo S (2018). Exosomes in myocardial repair: advances and challenges in the development of next-generation therapeutics. Mol Ther.

[39] Zhao T, Sun F, Liu J, Ding T, She J, Mao F, Xu W, Qian H, Yan Y (2019). Emerging role of mesenchymal stem cell-derived exosomes in regenerative medicine. Curr Stem Cell Res Ther.

[40] Maqsood M, Kang M, Wu X, Chen J, Teng L, Qiu L (2020). Adult mesenchymal stem cells and their exosomes: sources, characteristics, T and application in regenerative medicine. Life Sci.

[41] Lee AS, Tang C, Rao MS, Weissman IL, Wu JC (2013). Tumorigenicity as a clinical hurdle for pluripotent stem cell therapies. Nat Med.

[42] Han Y, Li X, Zhang Y, Han Y, Chang F, Ding J (2019). Mesenchymal stem cells for regenerative medicine. Cells.

[43] Chang YH, Wu KC, Harn HJ, Lin SZ, Ding DC (2018). Exosomes and stem cells in degenerative disease diagnosis and therapy. Cell Transplant.

[44] Yin K, Wang S, Zhao RC (2019). Exosomes from mesenchymal stem/stromal cells: a new therapeutic paradigm. Biomark Res.

[45] Zhang Y, Liu Y, Liu H, Tang WH (2019). Exosomes: biogenesis, biologic function and clinical potential. Cell Biosci.

[46] Maqsood M, Kang M, Wu X, Chen J, Teng L, Qiu L (2020). Adult mesenchymal stem cells and their exosomes: sources, characteristics, and application in regenerative medicine. Life Sci.

[47] Perocheau D, Touramanidou L, Gurung S, Gissen P, Baruteau J (2021). Clinical applications for exosomes: are we there yet?. Br J Pharmacol.

[48] McLean AC, Valenzuela N, Fai S, Bennett SA (2012). Performing vaginal lavage, crystal violet staining, and vaginal cytological evaluation for mouse estrous cycle staging identification. J Vis Exp.

[49] Cora MC, Kooistra L, Travlos G (2015). Vaginal cytology of the laboratory rat and mouse: review and criteria for the staging of the estrous cycle using stained vaginal smears. Toxicol Pathol.

[50] Funakoshi K, Bagheri M, Zhou M, Suzuki R, Abe H, Akashi H (2017). Highly sensitive and specific Alu-based quantification of human cells among rodent cells. Sci Rep.

[51] Hsu PL, Wang D, Ballard-Croft C, Xiao D, Zwischenberger JB (2017). A numerical simulation comparing a cavopulmonary assist device and VA ECMO for failing Fontan support. ASAIO J.

[52] Liu M, Zhang D, Zhou X, Duan J, Hu Y, Zhang W, Liu Q, Xu B, Zhang A (2022). Cell-free fat extract improves ovarian function and fertility in mice with premature ovarian insufficiency. Stem Cell Res Ther.

[53] Ishizuka B (2021). Current understanding of the etiology, symptomatology, and treatment options in premature ovarian insufficiency (POI). Front Endocrinol (Lausanne).

[54] Paciuc J (2020). Hormone therapy in menopause. Adv Exp Med Biol.

[55] Takahashi A, Yousif A, Hong L, Chefetz I (2021). Premature ovarian insufficiency: pathogenesis and therapeutic potential of mesenchymal stem cell. J Mol Med (Berl).

[56] Lai CP, Mardini O, Ericsson M, Prabhakar S, Maguire C, Chen JW, Tannous BA, Breakefield XO (2014). Dynamic biodistribution of extracellular vesicles in vivo using a multimodal imaging reporter. ACS Nano.

[57] Morishita M, Takahashi Y, Nishikawa M, Takakura Y (2017). Pharmacokinetics of exosomes—an important factor for elucidating the biological roles of exosomes and for the development of exosome-based therapeutics. J Pharm Sci.

[58] Kim DH, Kothandan VK, Kim HW, Kim KS, Kim JY, Cho HJ, Lee YK, Lee DE, Hwang SR (2019). Noninvasive assessment of exosome pharmacokinetics in vivo: a review. Pharmaceutics.

[59] Vizoso FJ, Eiro N, Cid S, Schneider J, Perez-Fernandez R (2017). Mesenchymal stem cell secretome: toward cell-free therapeutic strategies in regenerative medicine. Int J Mol Sci.

[60] LE Chen YS, Chiou TW, Harn HJ (2019). Exosomes in clinical trial and their production in compliance with good manufacturing practice. Tzu Chi Med J.

[61] Kordelas LRV, Ludwig AK, Radtke S, Ruesing J, Doeppner TR, Epple M, Horn PA, Beelen DW, Giebel B (2014). MSC-derived exosomes: a novel tool to treat therapy-refractory graft-versus-host disease. Leukemia.

[62] Nassar W, El-Ansary M, Sabry D, Mostafa MA, Fayad T, Kotb E, Temraz M, Saad AN, Essa W, Adel H (2016). Umbilical cord mesenchymal stem cells derived extracellular vesicles can safely ameliorate the progression of chronic kidney diseases. Biomater Res.

[63] Kwon HH, Yang SH, Lee J, Park BC, Park KY, Jung JY, Bae Y, Park GH (2020). Combination treatment with human adipose tissue stem cell-derived exosomes and fractional CO2 laser for acne scars: a 12-week prospective, double-blind, randomized, split-face study. Acta Dermato-Venereol.

[64] Jamshidi EBA, Soltani P, Niknejad H (2021). Proposed mechanisms of targeting COVID-19 by delivering mesenchymal stem cells and their exosomes to damaged organs. Stem Cell Rev Rep.

[65] Akbari A, Rezaie J (2020). Potential therapeutic application of mesenchymal stem cell-derived exosomes in SARS-CoV-2 pneumonia. Stem Cell Res.

[66] Sengupta V, Sengupta S, Lazo A, Hicok KC, Moseley T (2020). Exosomes derived from bone marrow mesenchymal stem cells as treatment for severe COVID-19. Stem Cells Dev.

[67] Mitrani MI, Bellio MA, Sagel A, Saylor M, Kapp W, VanOsdol K, Haskell G, Stewart D, Abdullah Z, Santos I (2021). Case report: administration of amniotic fluid-derived nanoparticles in three severely Ill COVID-19 patients. Front Med.

