# Supplementary material for: Comparison of the therapeutic effects between stem cells and exosomes in primary ovarian insufficiency: as promising as cells but different persistency and dosage
Source: Stem Cell Res Ther. 2023 Jun 20;14:165. doi: 10.1186/s13287-023-03397-2 (PMC10283237; doi:10.1186/s13287-023-03397-2)
Supplement: Supplementary file 2 — Additional file 2. Supplementary data2_List of differentially expressed genes. [file 13287_2023_3397_MOESM2_ESM.pdf]

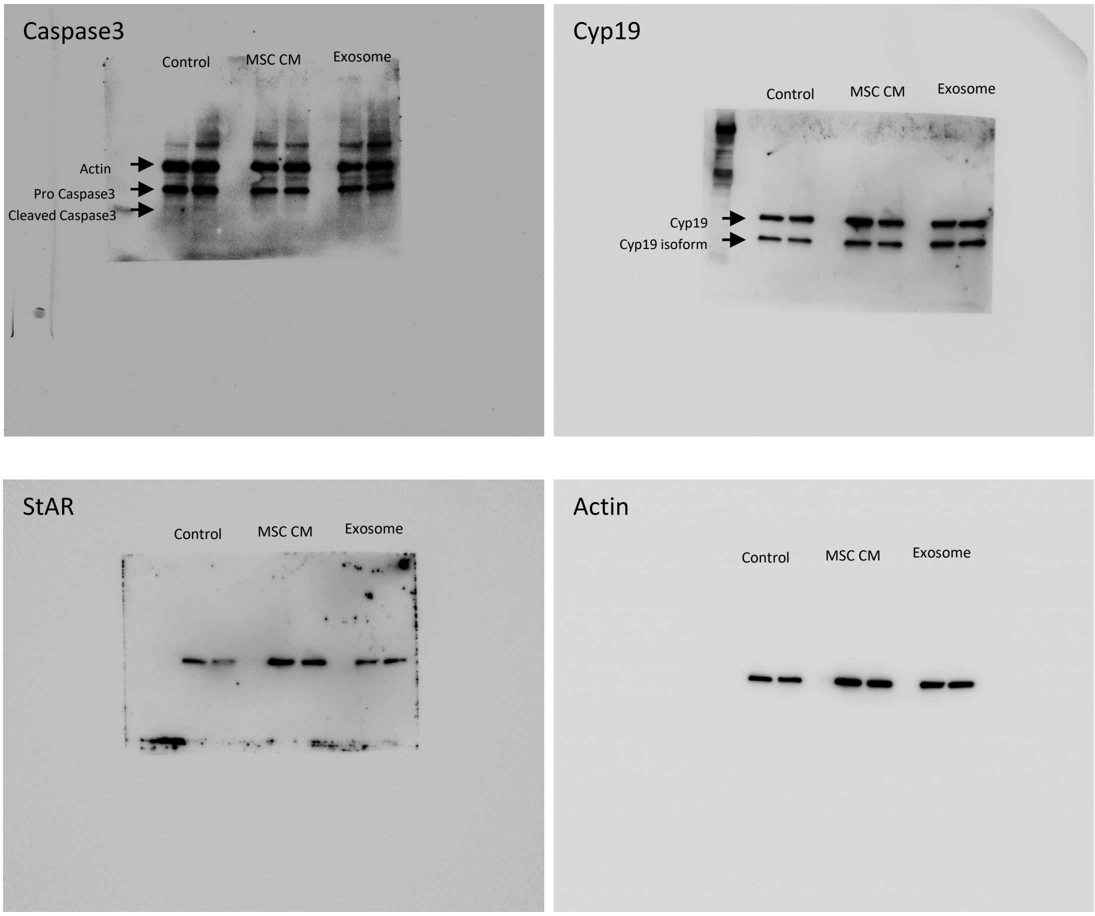


Supplementary figure 1. Original  uncropped blot images. Protein were corrected from HGrC1 cells after 24 hours of treatment with MSC CM and exosomes.
