# Supplementary material for: Comparison of the therapeutic effects between stem cells and exosomes in primary ovarian insufficiency: as promising as cells but different persistency and dosage
Source: Stem Cell Res Ther. 2023 Jun 20;14:165. doi: 10.1186/s13287-023-03397-2 (PMC10283237; doi:10.1186/s13287-023-03397-2)
Supplement: Supplementary file 3 — Additional file 3. Original blot images. [file 13287_2023_3397_MOESM3_ESM.docx]

|  |  | Cycle length (days) | Extended cycle  (>95% CI) | Estrus stage frequency |
| --- | --- | --- | --- | --- |
| Control mice | control #1 | 5 | - | 2 |
|  | control #2 | 5 | - | 2 |
|  | control #3 | 4 | - | 1 |
|  | control #4 | 5 | - | 2 |
|  | control #5 | 5 | - | 2 |
|  | control #6 | 5 | - | 2 |
| Confidence interval (CI) of control | Lower 95% CI of mean | 4.586 |  |  |
|  | Upper 95% CI of mean | 5.081 |  |  |
| Untreated POI mice | POI #1 | 8 | Yes | 0 |
|  | POI #2 | 8 | Yes | 0 |
|  | POI #3 | 8 | Yes | 0 |
|  | POI #4 | 8 | Yes | 0 |
|  | POI #5 | 8 | Yes | 0 |
|  | POI #6 | 8 | Yes | 0 |
| MSC treated POI mice | MSC-10K #1 | 3 | - | 1 |
|  | MSC-10K #2 | 8 | Yes | 0 |
|  | MSC-10K #3 | 8 | Yes | 0 |
|  | MSC-10K #4 | 8 | Yes | 0 |
|  | MSC-10K #5 | 5 | - | 1 |
|  | MSC-100K #1 | 8 | Yes | 0 |
|  | MSC-100K #2 | 8 | Yes | 0 |
|  | MSC-100K #3 | 8 | Yes | 0 |
|  | MSC-100K #4 | 8 | Yes | 0 |
|  | MSC-100K #5 | 5 | - | 2 |
|  | MSC-1M #1 | 6 | Yes | 2 |
|  | MSC-1M #2 | 8 | Yes | 0 |
|  | MSC-1M #3 | 8 | Yes | 0 |
|  | MSC-1M #4 | 6 | Yes | 1 |

Supplementary table 1. Estrus cycle analysis for 8 days after MSC treatment

|  |  | Cycle length (days) | Extended cycle  (>95% CI) | Estrus stage frequency |
| --- | --- | --- | --- | --- |
| Control mice | control #1 | 4 | - | 2 |
|  | control #2 | 4 | - | 2 |
|  | control #3 | 4 | - | 2 |
|  | control #4 | 3 | - | 2 |
|  | control #5 | 6 | Yes | 1 |
| Confidence interval (CI) of control | Lower 95% CI of mean | 3.332 |  |  |
|  | Upper 95% CI of mean | 5.405 |  |  |
| Untreated POI mice | POI #1 | 8 | Yes | 0 |
|  | POI #2 | 8 | Yes | 0 |
|  | POI #3 | 8 | Yes | 0 |
|  | POI #4 | 8 | Yes | 0 |
|  | POI #5 | 7 | Yes | 0 |
| MSC treated POI mice | UC-Exo #1 | 6 | Yes | 1 |
|  | UC-Exo #2 | 7 | Yes | 1 |
|  | UC-Exo #3 | 8 | Yes | 1 |
|  | UC-Exo #4 | 7 | Yes | 2 |
|  | UC-Exo #5 | 4 | - | 2 |
|  | BM-Exo #1 | 8 | Yes | 0 |
|  | BM-Exo #2 | 8 | Yes | 0 |
|  | BM-Exo #3 | 4 | - | 1 |
|  | BM-Exo #4 | 8 | Yes | 0 |

Supplementary table 2. Estrus cycle analysis for 8 days after exosome treatment
